# The Influence of the Annealing Process on the Mechanical Properties of Chromium Nitride Thin Films

**DOI:** 10.3390/ma18153605

**Published:** 2025-07-31

**Authors:** Elena Chițanu, Iulian Iordache, Mirela Maria Codescu, Virgil Emanuel Marinescu, Gabriela Beatrice Sbârcea, Delia Pătroi, Leila Zevri, Alexandra Cristiana Nadolu

**Affiliations:** 1 R&D National Institute for Electrical Engineering ICPE-CA, 030138 Bucharest, Romania; 2 “Alexandru Ioan Cuza” National Military College, 900234 Constanta, Romania; 3 Faculty of Engineering in Foreign Languages, National University of Science and Technology POLITEHNICA, 060042 Bucharest, Romania

**Keywords:** chromium nitride, Cr_2_O_3_, thin films, reactive magnetron sputtering, oxidation

## Abstract

In recent years, significant attention has been directed toward the development of coating materials capable of tailoring surface properties for various functional applications. Transition metal nitrides, in particular, have garnered interest due to their superior physical and chemical properties, including high hardness, excellent wear resistance, and strong corrosion resistance. In this study, a fabrication process for CrN-based thin films was developed by combining reactive direct current magnetron sputtering (dcMS) with post-deposition annealing in air. CrN coatings were deposited by reactive dcMS using different argon-nitrogen (Ar:N_2_) gas ratios (4:1, 3:1, 2:1, and 1:1), followed by annealing at 550 °C for 1.5 h in ambient air. XRD and EDS analysis revealed that this treatment results in the formation of a composite phase comprising CrN and Cr_2_O_3_. The resulting coating exhibited favorable mechanical and tribological properties, including a maximum hardness of 12 GPa, a low wear coefficient of 0.254 and a specific wear rate of 7.05 × 10^−6^ mm^3^/N·m, making it a strong candidate for advanced protective coating applications.

## 1. Introduction

In recent years, thin film coatings have garnered significant attention due to their ability to tailor the surface properties of structural materials for advanced mechanical and tribological applications. The performance of such coatings is strongly influenced by a combination of factors, including the intrinsic properties of the coating materials, the deposition technique employed and the physical and chemical characteristics of the substrate [1]. By carefully selecting material systems and optimizing the coating architecture, thin films can be engineered to reduce friction and improve wear resistance in a variety of operating environments [2,3]. Owing to their high structural versatility, thin film systems continue to attract significant research attention aimed at optimizing performance and ensuring long-term stability under extreme service conditions [4]. Nevertheless, since these coatings are frequently exposed to oxidative environments at elevated temperatures during operation, oxidation resistance has become a critical factor for their practical applicability, directly influencing their reliability and long-term performance under demanding conditions.

Friction and wear represent major challenges for the performance and longevity of mechanical system components, often leading to excessive energy consumption and progressive material degradation. It is estimated that up to 20–25% of industrial energy is lost due to friction in processes such as paper/pulp production and mining, which corresponds to around 20% of total industrial energy use [5]. Studies indicate that the application of thin hard coatings, such as PVD-deposited metal nitrides, can reduce friction and wear-related losses by approximately 10–15%, thereby extending the operational lifespan of mechanical parts [5]. In sliding applications, coatings made from high-hardness, wear-resistant materials (e.g., TiN, DLC) not only increase service life, but also reduce manufacturing and maintenance costs [6]. Moreover, thin film coatings can be engineered to provide unique or multifunctional surface characteristics, such as low friction, corrosion resistance or solid lubrication, which enhance tribological performance in diverse environments.

Thin film coatings can be fabricated using a wide range of deposition techniques, including electrochemical methods, chemical vapor deposition (CVD), and physical vapor deposition (PVD). Among these, electrochemical deposition is the most commonly employed method for producing chromium-based coatings; in the case of CrN, the process involves, in addition to the electrochemical deposition of a chromium precursor layer, a subsequent heat treatment in a nitrogen atmosphere, at high temperatures (up to 1000 °C), necessary for the formation of the chromium nitride (CrN) phase [7]. This electrochemical process involves the use of chromic acid solutions containing hexavalent chromium (Cr(VI)), a compound known for its high toxicity and severe environmental and health hazards [8]. In response to these concerns, the European Union has implemented regulations to restrict the use of Cr(VI), most notably through the Waste Electrical and Electronic Equipment (WEEE) Directive and the Restriction of Hazardous Substances (RoHS) Directive [9,10]. Similarly, the United States Environmental Protection Agency (EPA) has classified hexavalent chromium as a Group A carcinogen, indicating that it is carcinogenic to humans based on sufficient evidence from epidemiological studies [11].

Chemical vapor deposition and physical vapor deposition are dry deposition techniques that comply with European regulations restricting hexavalent chromium usage. CVD is particularly suited for producing coatings with high adhesion and desirable mechanical and tribological properties. However, CVD typically requires substrate temperatures well above 300 °C, commonly ranging from 750 °C to 1000 °C in conventional thermal processes. This limits its applicability to substrates that can withstand such elevated temperatures without undergoing thermal degradation [12]. PVD methods, such as sputtering, operate at significantly lower temperatures compared to CVD. In sputtering, atoms are ejected from a solid target through ion bombardment, typically by argon ions (Ar^+^) and subsequently deposited onto the substrate under vacuum or low-pressure gas conditions [13].

Sputtering processes allow for control over deposition parameters, enabling the tailoring of film properties such as residual stress, hardness, surface morphology and microstructure to meet the demands of structural and functional applications. This versatility makes sputtering a preferred technique in the development of high-performance thin films [14,15]. Among the various sputtering methods, dcMS is commonly employed for the deposition of metallic coatings. In dcMS, a direct current is applied to a metallic target and a magnetic field, typically generated by permanent magnets positioned behind the target, and serves to confine the plasma in close proximity to the target surface. This magnetic confinement increases the ionization efficiency of the working gas (usually argon), enhances the deposition rate and ensures more uniform film growth [16,17]. Substrate heating is a critical factor influencing thin film growth, as it enhances adatom mobility and thereby improves film crystallinity and density in reactive dcMS. However, if the temperature is not properly balanced with other deposition parameters, it can lead to defects such as voids, abnormal grain growth or stress-induced dislocations [18].

Transition metal nitrides are widely used in engineering applications due to their exceptional physical and chemical properties [19], including high hardness, excellent wear resistance, thermal stability, and strong resistance to corrosion and oxidation. In recent decades, significant research has been devoted to the development and characterization of such nitrides as functional thin film coatings.

Notable examples include titanium nitride (TiN) [20], CrN [21], zirconium nitride (ZrN) [22], molybdenum nitride (MoN) [23], niobium nitride (NbN) [24], and tantalum nitride (TaN) [25]. The growing demand for high-performance protective and functional coatings in industrial sectors such as tooling, aerospace, and electronics has driven the advancement of deposition techniques and the optimization of these nitride-based thin films. When selecting an appropriate coating material, several parameters must be carefully considered, including the intrinsic hardness of the material, its crystal structure and the chemical compatibility between the coating and the substrate. These factors collectively influence the adhesion, durability, and overall effectiveness of the coating in demanding service environments.

While certain nitrides demonstrate superior mechanical and thermal properties relative to chromium nitride (e.g., TiN, AlN) [26], this study focuses on CrN to develop a dry thin-film deposition technique as an environmentally benign alternative to conventional electrochemical methods that rely on hexavalent chromium-containing solutions [27,28]. CrN films are widely employed as protective coatings to extend the service life of cutting tools and machine components, owing to their high hardness, excellent adhesion to substrate materials and superior resistance to wear and corrosion [29]. However, since these coatings are often exposed to oxidative environments at elevated temperatures during operation, oxidation resistance has become increasingly important for their practical application.

This study investigates the influence of nitrogen supply in the deposition chamber on the structural, morphological, and functional characteristics of CrN coatings prepared using reactive dcMS with varying Ar:N_2_ gas ratios. The coatings were produced using a “dry” deposition technique, representing an environmentally friendly alternative to traditional electrochemical chroming methods, which involve the use of hexavalent chromium, known to be highly toxic to both humans and the environment. Additionally, the effect of post-deposition annealing in air on the structural, mechanical and tribological properties of the CrN-based coatings was systematically examined.

## 2. Materials and Methods

### 2.1. Thin Films Deposition

CrN thin films were deposited using the reactive dcMS method. High-purity chromium targets (99.99%) with a diameter of 2 inches and a thickness of 6 mm (Ottamagation, Tallinn, Estonia) served as the sputtering source. The deposition was carried out in a vacuum chamber with a base pressure of 8.5 × 10^−5^ mbar. The substrates were rotated at 30 rpm and maintained at a temperature of 300 °C throughout the deposition. The target-to-substrate distance was fixed at 20 cm for all prepared films. Prior to deposition, substrates, including NBK-7 glass, silicon wafers, and stainless-steel type 304L disks with a diameter of 2.4 cm, were cleaned ultrasonically for 10 min sequentially in acetone, ethanol, and deionized water, followed by drying with argon gas. For the reactive dcMS deposition of CrN argon (Ar) as the working gas and nitrogen (N_2_) as the reactive gas were used, with varying gas flow ratios of Ar:N_2_ at 1:1, 2:1, 3:1, and 4:1 (denoted as samples CrN1/1, CrN2/1, CrN3/1, and CrN4/1, respectively). The sputtering was performed with a power density of 12.34 W/cm^2^ applied to the chromium target, at a deposition pressure of 2.2 Pa, for a duration of 45 min. Following deposition, the CrN coatings were subjected to post-deposition annealing in air at 550 °C for 1.5 h.

### 2.2. Characterization

The structural characterization of chromium nitride films was performed using the D8 Discover diffractometer (BRUKER AXS GmbH, Germany) with Cu Kα radiation, (λ = 1.540598 Å), operated at a voltage of 40 kV, intensity current of 40 mA, using a scintillation detector. The diffractograms were recorded in 2*θ* angular range of 30–100° and incidence angle of 2°.

The surface morphology and thickness of thin film coatings were observed by FESEM-FIB Scanning Electron Microscope (Workstation Auriga), equipped with a dispersive X-ray spectometer (Oxford Instruments) model X-MAXn type, and AZTEC software. The average roughness and root mean square (RMS) roughness were evaluated together with the surface morphology by a Semi-contact AFM technique on SPM Ntegra Aura (NT-MDT, Russia) equipment, using a NSG30_SS (NT-MDT, Russia), Golden Silicon Probe with a curvature radius of 2 nm. Data acquisition and processing were performed using Nova v.1.0.26 (NT-MDT, Russia) software. The average roughness and root mean square (RMS) roughness are defined as follows:

$$R_{a}=\frac{1}{n}\sum_{i=1}^{n}\left|y_{i}\right|\;\mathrm{and}\;R_{q}=\sqrt{\sum_{i=1}^{n}y_{i}^{2}}$$

where y is the height (z) at a given pixel (i) in the image.

Tribological tests of CrN coatings were conducted at room temperature using a ball-on-disk tribometer with a rotating module (CSM Instruments, Peseux, Switzerland) in accordance with the ASTM G99-17 standard [30]. A constant normal load (F_n_) of 0.5 N was applied to the sample via a static partner. For the as-deposited CrN films, a 100Cr6 steel ball (Vickers hardness HV_10_ = 850, Young’s modulus = 210 GPa) was used, while for the annealed films, an Al_2_O_3_ ball (Vickers hardness HV_10_ = 1700, Young’s modulus = 390 GPa) was used. In both cases, the static partner had a diameter of 6 mm and followed a circular sliding path with a radius of 6 mm, resulting in a total sliding distance of 37.68 m at a constant linear velocity of 10 cm/s. The deflection of the static partner during testing was measured and recorded as the tangential load (F_t_). The coefficient of friction (µ) was determined with Equation (1), using the ratio between F_t_ and F_n_ [31]

μ = F_t_/F_n_ (1)

The modification of friction coefficient-COF (µ) was registered versus sliding distance using TriboX 4.1.I software. The specific wear rate (W_s_, in m^3^/(N·m), was calculated with Equation (2) [31]

W_S_ = V/(F_n_ D) (2)

where *V* is the removed material from the track in (mm^3^), *F_n_* is the normal load (N), and *D* is the sliding distance (m) performed during the tribological test.

Mechanical tests were performed with a nanoindentation module (NHT^2^) using a diamond Berkovich indenter installed on the Micro-Combi Tester platform (CSM Instruments, Peseux, Switzerland). In the case of CrN coatings and the SS304L substrate (Ø2.4 cm × 1 mm), nanoindentation testing with the Indentation 4.37 software and the Oliver and Pharr calculation method were used [32], according to ISO 14577-1:2015 [33] and ISO 14577-4:2016 [34] standards. For each sample ten tests (advanced mode) were completed, with load control for the SS304L substrate and depth control (maximum depth of 1/10 of coating thickness) for the CrN coatings. The measurement conditions are shown in Table 1.

## 3. Results and Discussion

### Chromium Nitride Thin Films

CrN thin films were prepared using the reactive dcMS technique, which involves the use of a reactive gas, typically nitrogen, alongside a working gas such as argon. In this process, argon ions are accelerated toward the chromium target, causing the ejection (sputtering) of chromium atoms. Simultaneously, the introduced nitrogen gas reacts with the sputtered chromium atoms, forming CrN. This compound is subsequently deposited as a thin film on the substrate surface, resulting in the formation of a CrN coating.

CrN thin films were deposited using reactive dcMS to investigate the influence of the reactive gas flow rate on their structural, morphological, mechanical, and tribological properties. Argon-to-nitrogen (Ar:N_2_) gas flow ratios of 4:1, 3:1, 2:1, and 1:1 were utilized, corresponding to samples designated as CrN4/1, CrN3/1, CrN2/1, and CrN1/1, respectively.

**Figure 1:**
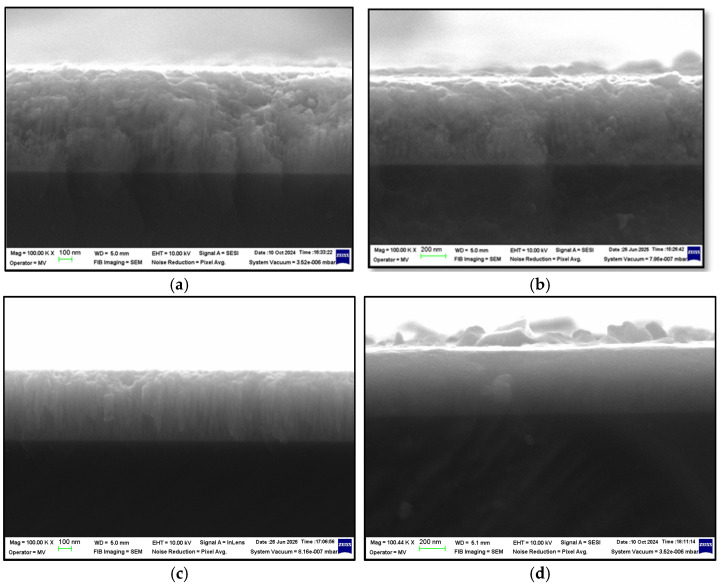
Cross-sectional SEM images of CrN thin films as-deposited on silicon substrates at different Ar:N_2_. gas flow ratios: (**a**) CrN4/1, (**b**) CrN3/1, (**c**) CrN2/1, and (**d**) CrN1/1 (magnification: 100 K×).

Scanning Electron Microscopy analysis was conducted on the as-deposited CrN thin films on silicone substrate to determine coating thickness (Figure 1). Based on the measured thickness values, the deposition rate was calculated and found to decrease with nitrogen content (Table 2). Initially, for an Ar:N_2_ ratio of 4:1, the deposition rate was 17.88 nm/min, which slightly decreased to 17.77 nm/min at a 3:1 ratio. A more noticeable reduction was observed at a 2:1 ratio, with a deposition rate of 15.11 nm/min, reaching a minimum of 11.11 nm/min at an equal Ar:N_2_ ratio of 1:1. The reduction in deposition rate with increasing nitrogen content in the chamber may be attributed to additional reactions occurring at the Cr target surface, leading to target poisoning. Additionally, the higher nitrogen concentration increases the frequency of collisions between sputtered particles and gas atoms, thereby reducing the number of chromium atoms that successfully reach the substrate [36].

**Figure 2:**
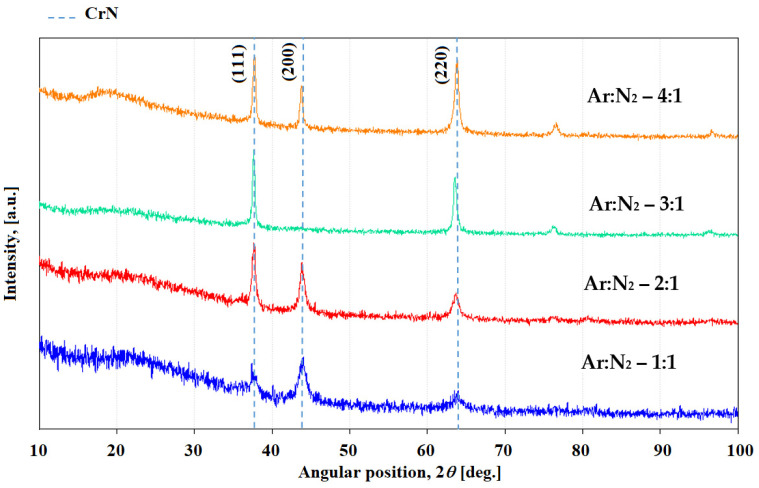
Comparative XRD diffractograms of CrN thin films deposited on NBK-7 glass substrates using different Ar:N_2_ gas flow ratios.

The comparative X-ray diffraction patterns of CrN thin films deposited on NBK-7 glass substrates at different Ar:N_2_ ratios (4:1, 3:1, 2:1, and 1:1) are presented in Figure 2. The XRD spectra exhibit three main peaks corresponding to the cubic crystalline structure of CrN (JCPDS Card No.: 076-2494, cubic system, space group Fm-3m, no. 225). The peaks observed at 2θ values of approximately 37°, 43°, and 64.5° are indexed to the (111), (200), and (220) crystallographic planes of CrN, respectively. These results indicate the presence of polycrystalline growth with no strong preferential orientation along a specific crystallographic direction. Importantly, no peaks corresponding to chromium oxide phases were detected. The characteristics of the dominant XRD peaks change with increasing nitrogen content: the peak positions shift slightly, and the peak shapes broaden as indicated by an increase in the full width at half maximum (FWHM).

The influence of varying Ar:N_2_ gas ratios on the structural characteristics of the deposited CrN films is illustrated in Figure 2. The X-ray diffraction patterns reveal that modifying the nitrogen content in the sputtering atmosphere enhances crystallographic growth along the (220) and (111) planes. This preferential orientation suggests that nitrogen plays a key role in promoting specific crystallographic textures during film deposition.

**Figure 3:**
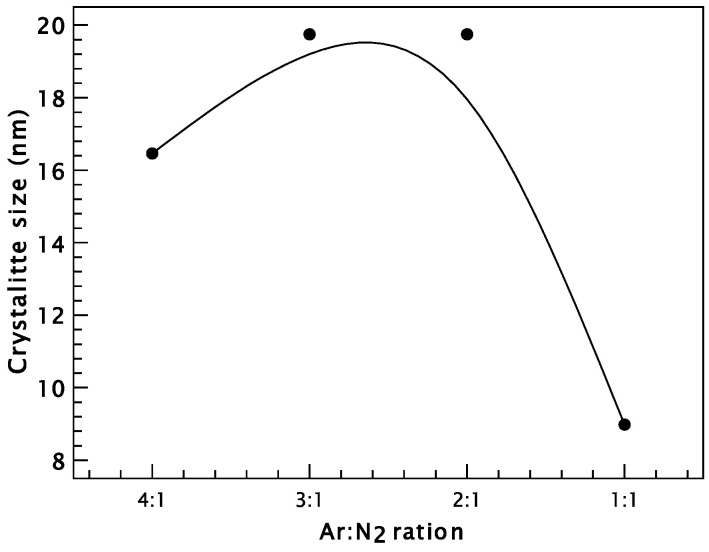
Variation in crystallite sizes of CrN thin films deposited on NBK-7 glass substrates at different Ar:N_2_ gas ratios.

The film deposited with the lowest N_2_ content exhibits multiple crystallographic orientations, with a dominant texture along the [220] direction, indicative of a textured microstructure. This behavior is likely driven by the predominance of strain energy over surface free energy during deposition, which typically favors growth along the (111) plane [37]. Furthermore, as the nitrogen content increases, the XRD peaks progressively broaden, particularly at an Ar:N_2_ ratio of 1:1, suggesting a decrease in average crystallite size (Figure 3), likely due to increased lattice distortion and limited grain growth under nitrogen-rich conditions [38].

X-ray diffraction analysis of the CrN thin films revealed a highly defective crystalline structure, with dislocation densities reaching values as high as 10^16^ lines/m^2^ (Table 3). This high density of defects can be attributed to the conditions present during deposition via reactive dcMS on heated substrates. The elevated substrate temperature (300 °C), combined with the energetic bombardment of incident particles, leads to enhanced adatom mobility and the generation of significant internal stresses, both of which contribute to the formation of a defect-rich microstructure [39].

To mitigate these defects within the film, a post-deposition heat treatment was performed in an air atmosphere. The annealing temperature was carefully selected to be sufficiently low to prevent the complete oxidation of the CrN film, while being high enough to facilitate favorable modifications to its microstructure. Considering these factors and supported by literature reports that demonstrate the oxidation stability of CrN up to 600 °C [40], the heat treatment was conducted at 550 °C. Annealing at 550 °C often allows the recrystallization or grain growth in CrN films without causing phase decomposition or excessive oxidation. This temperature can improve the crystallinity and reduce defects in the film, enhancing mechanical and physical properties like hardness and adhesion. CrN tends to oxidize at elevated temperatures in air, but 550 °C is often chosen because it is high enough to improve the film structure but not so high that severe oxidation occurs immediately. It allows for controlled oxidation, possibly forming a thin protective oxide layer (like Cr_2_O_3_), which can enhance corrosion resistance.

The crystallite size was calculated at the main peak (111) using Scherrer’s formula [41] and the results are presented in Table 3 and its variation with the gas flow ratio in Figure 3. It was found that the nanostructured coatings processed with the lowest nitrogen content have a crystallite size of 16.46 nm. Furthermore, for the CrN film deposited with an Ar:N_2_ gas ratio of 3:1, an increase in crystallite size up to 19.75 nm is observed, a value that is maintained even for the gas ratio of 2:1. When the nitrogen flow rate increases, it is found that the crystallite size increases, which can be attributed to the fact that at a higher N_2_ flow rate, the plasma expansion is lower and therefore the mobility of the reactants decreases and simultaneously the particle density increases [42]. The mean free path of the gas particles is shorter and decreases with an increasing nitrogen flow rate. The decrease in the mean free path also means a smaller source of energy and momentum on the substrate from the ion impact. This leads to a smaller extent of surface damage and the decreasing nucleation sites, which can lead to a larger grain size [43]. Then, with increasing nitrogen supply, the (111) peaks are broadened and crystallite size decreases to values of 8.98 nm.

**Figure 4:**
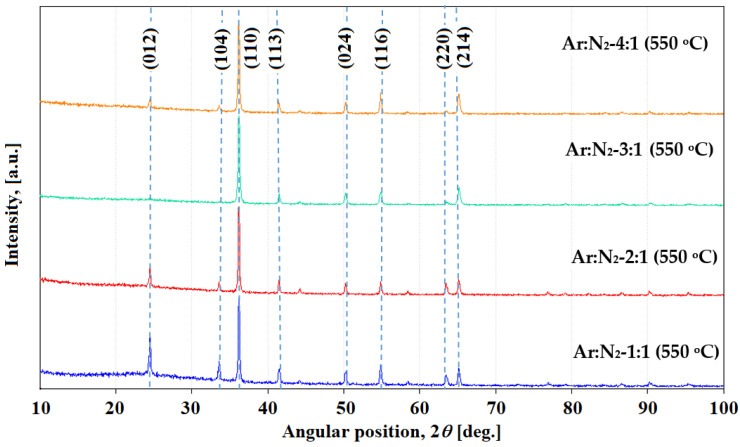
Comparative XRD diffractograms of CrN thin films deposited on NBK-7 glass substrates at different Ar:N_2_ gas ratios and subsequently annealed at 550 °C for 1.5 h.

XRD analysis was also performed on the annealed samples at a temperature of 550 °C for 1.5 h and the effect of the heat treatment on the structural characteristics of the deposited CrN film was determined using the X-ray patterns in Figure 4.

The transformation from the c-CrN phase to the r-Cr_2_N phase during air annealing is due to nitrogen depletion and subsequently both phases decompose with the formation of chromium oxide due to heat treatment at 550 °C [44,45], resulting in a mixture of CrN and Cr_2_O_3_. But Lu et al.’s study found that Cr_2_N is not thermodynamically stable at temperatures below 1010 °C, based on Gibbs free energy calculations; this is not obtained in the composition of the annealed film [46]. At elevated temperatures, such as 550 °C, coatings with a high concentration of structural defects exhibit accelerated diffusion processes that significantly alter their chemical and structural stability. Oxygen from the surrounding atmosphere diffuses inward through these defects, leading to the internal oxidation of metallic components within the coating matrix. Concurrently, nitrogen and chromium exhibit outward diffusion, further promoting the decomposition of nitride phases and the formation of stable oxides at or near the surface. This transformation can be described by the following reactions [46]:

2 CrN → Cr_2_N + 1/2 N_2_ (3)

Cr_2_N → 2 Cr + 1/2 N_2_ (4)

2 CrN + 3/2 O_2_ → Cr_2_O_3_ + N_2_ (5)

Cr_2_N + 3/2 O_2_ → Cr_2_O_3_ + 1/2 N_2_ (6)

Reactions (5) and (6) occur at the film surface, where nitrogen is released as N_2_ due to its volatility. Within the film bulk, reactions (3) and (4) proceed in the presence of oxygen, leading to nitrogen elimination from nitrides and the formation of oxide phases.

X-ray diffraction analysis confirmed the formation of Cr_2_O_3_ and CrN phases in the coatings after heat treatment at 550 °C in air. The Cr_2_O_3_ phase was identified by characteristic diffraction peaks at 2θ values of 24.5° (012), 33.6° (104), 36.2° (110), 41.5° (113), 50.2° (024), 54.8° (116), and 65.18° (214), consistent with the standard reference pattern (JCPDS Card No. 38-1479). The presence of CrN was confirmed by the diffraction peak located at 63.5°, which corresponds to the (220) plane, in agreement with JCPDS Card No. 76-2494. These results indicate that annealing at 550 °C in an oxidizing environment leads to the partial oxidation of CrN and the formation of a composite structure comprising both CrN and Cr_2_O_3_ phases.

**Figure 5:**
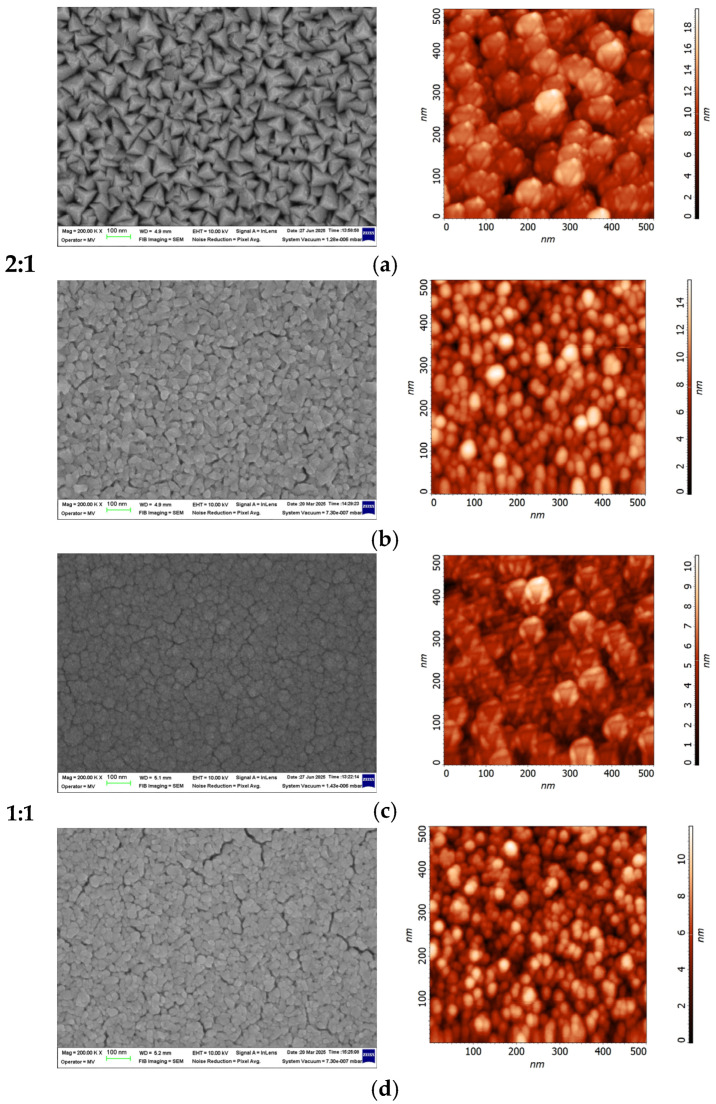
Comparative SEM images and AFM surface morphologies of CrN thin films deposited on silicone substrates under varying Ar:N_2_ gas ratios: (**a**) 2:1 as-deposited, (**b**) 2:1 annealed at 550 °C, (**c**) 1:1 as-deposited, and (**d**) 1:1. annealed at 550 °C.

**Figure 6:**
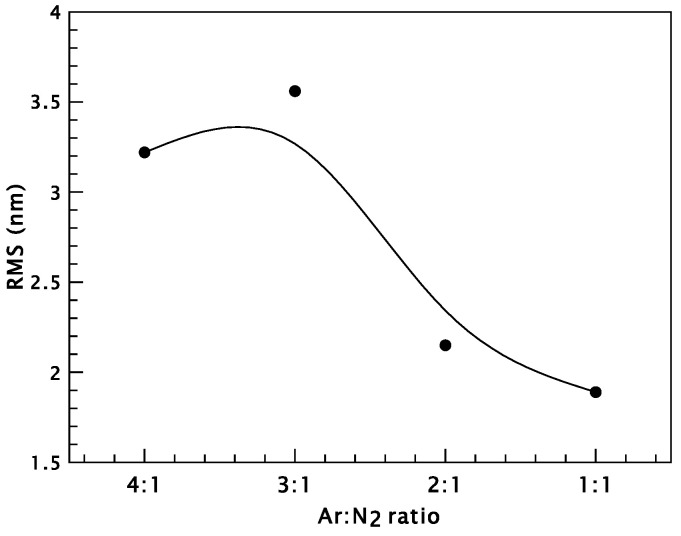
Root mean square surface roughness of CrN deposited on silicone substrates and annealed at 550 °C as a function of the Ar:N_2_ gas ratio during deposition.

The surface morphologies of the CrN thin film deposited on silicone substrates were characterized using atomic force microscopy (AFM) and scanning electron microscopy (SEM). A comparative analysis between the as-deposited and annealed films is presented in Figure 5. The root mean square (RMS) roughness values of the CrN coatings are presented in Figure 6.

The RMS roughness of as-deposited coatings exhibits lower values compared to the annealed samples. The RMS roughness ranges from 3.20 nm for films deposited at an Ar:N_2_ ratio of 4:1, to 2.87 nm for 3:1, decreasing further to 2.02 nm for 2:1, and reaching a minimum of 1.5 nm at the 1:1 ratio. After annealing, the roughness values increase across all samples, indicating surface morphology changes induced by the heat treatment [47]. For the annealed samples, increasing the nitrogen content from an Ar:N_2_ ratio of 4:1 to 3:1 initially causes the surface roughness to increase from 3.22 nm to 3.56 nm. However, further increases in nitrogen content at ratios of 2:1 and 1:1 result in a gradual decrease in roughness, reaching 2.15 nm and a minimum of 1.89 nm, respectively.

In the SEM images shown in Figure 5 for films deposited at Ar:N_2_ ratios of 2:1 and 1:1, a comparison between as-deposited and annealed samples reveals notable changes in surface morphology with increasing nitrogen content. These changes are attributed, on one hand, to modifications in crystallite size and inter-grain spacing caused by the increased nitrogen supply during deposition and on the other hand, to the effects of the annealing treatment. These surface modifications corroborate the AFM analysis results, which show a decrease in surface roughness with increasing nitrogen content during deposition. The enlarged inter-grain spaces facilitate oxidation by enabling nitrogen release and oxygen ingress into the coating. Moreover, the observed changes in surface morphology are consistent with the XRD analysis.

Subsequently, energy-dispersive X-ray spectroscopy analysis was conducted on both the as-deposited CrN films and the annealed samples to investigate their elemental composition and any changes induced by thermal treatment.

Oxygen detected by EDS in as-deposited CrN films, reaching up to 20.2 at. % (Table 4) is most likely the result of partial oxidation occurring during deposition and subsequent exposure to atmospheric oxygen. Similarly, oxygen levels in annealed films, which increase up to 56.2 at. % (Table 5), are attributed to residual oxidation from deposition, environmental contamination, and oxygen diffusion during thermal treatment [48]. Consequently, oxygen may be incorporated both as an impurity and through the formation of Cr_2_O_3_. Due to the reduced thickness of the films, silicon was also detected in the EDS analysis, which corresponds to the substrate material used.

**Figure 7:**
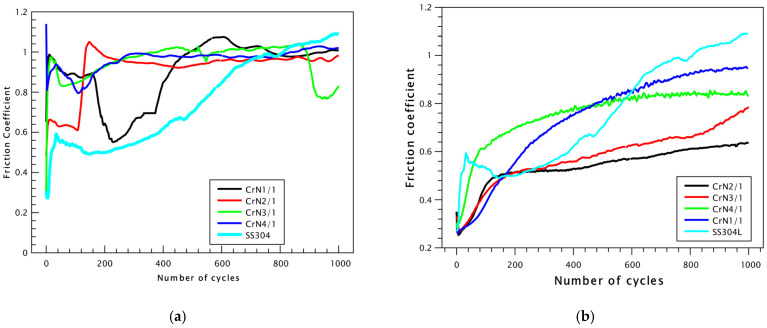
Friction coefficient curves of as-deposited CrN coatings deposited on SS304L stainless-steel substrates (**a**) and 550 °C annealed coatings (**b**).

The frictional and wear behavior of the CrN coatings deposited on SS304L stainless-steel substrates was systematically investigated. Figure 7 illustrates the COF curves obtained from sliding tests. For the as-deposited CrN coatings (Figure 7a), tests were performed on an SS304L stainless-steel substrate using a Cr100 static counterpart under dry sliding conditions. Conversely, for the CrN coatings annealed at 550 °C (Figure 7b), an Al_2_O_3_ ball was employed as the static counterpart to evaluate frictional performance under identical conditions.

**Figure 8:**
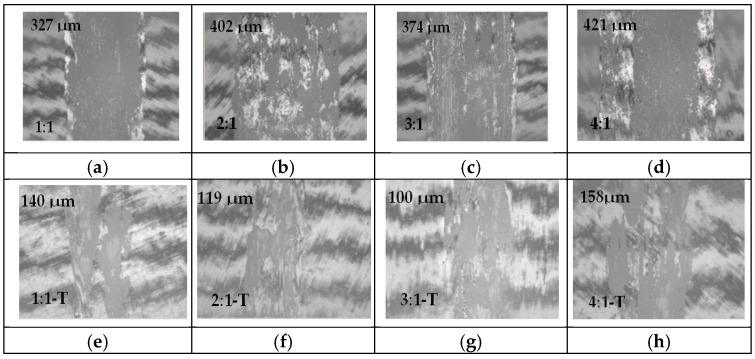
Comparative optical micrographs of the wear tracks for as-deposited CrN coatings deposited on SS304L stainless-steel substrates (**a**–**d**) and annealed coatings (**e**–**h**) corresponding to different Ar:N_2_ gas ratios (magnification ×10).

Figure 7 illustrates that annealing the CrN coating has a direct effect on the COF, which correlates with a reduction in the wear track by approximately 50%, as shown in Figure 8. For CrN coatings, the COF decreases as the nitrogen input decreases. Specifically, the highest COF value of 0.963 is observed for an Ar:N_2_ gas ratio of 1:1. When the gas ratio is adjusted to 2:1, the COF decreases slightly to 0.956, while the lowest COF values of 0.919 and 0.918 are recorded for ratios of 3:1 and 4:1, respectively. Figure 7a shows that all CrN coatings exhibit higher COF values compared to the stainless-steel substrate (indicated by the light blue line). After annealing at 550 °C, the COF values significantly decrease compared to the as-deposited CrN coatings. The COF reaches 0.325 for the Ar:N_2_ ratio of 1:1 and achieves the lowest value of 0.254 for the ratio of 2:1. For gas ratios of 3:1 and 4:1, higher COF values of 0.459 and 0.401 are observed, respectively.

The improvement of the tribological properties of the substrate was evaluated using pin-on-disk testing. All annealed coatings demonstrated lower COF compared to the bare substrate for up to approximately 100 cycles. Upon extended testing to 300 cycles, the annealed coating deposited with an Ar:N_2_ gas ratio of 2:1 exhibited the lowest COF value of 0.254, which is notably lower than that recorded for the stainless-steel substrate. Figure 7b illustrates that all CrN coatings exhibit lower COF compared to the stainless-steel substrate, as indicated by the light blue line.

After performing the pin-on-disk tribological tests, the wear tracks recorded with the 100Cr6 steel ball for as-deposited CrN films were analyzed; traces that show different widths and pieces of the transfer layer were visible on its surface. In the case of CrN coatings, the wear track is uniform and it can be seen that the width of the trace is influenced by the ratio of working and reactive gases. Thus, for a ratio of 1:1 a width of 327 µm is recorded, for a ratio of 2:1 the trace increases to a value of 402 µm, decreases to value of 374 for a gas ratio of 3:1 and for the lowest nitrogen supply a trace with a width of 421 µm is achieved, as can be seen from Figure 8a–d.

After annealing at 550 °C, the wear tracks produced by the Al_2_O_3_ ball were analyzed. The coatings exhibited non-uniform wear tracks with irregular surfaces, and the track widths were visibly reduced, approximately half the size, compared to those of the as-deposited CrN coatings, as shown in Figure 8e–h. The wear track widths ranged from a minimum of 100 µm for an Ar:N_2_ gas ratio of 3:1 to a maximum of 158 µm for a ratio of 4:1.

**Figure 9:**
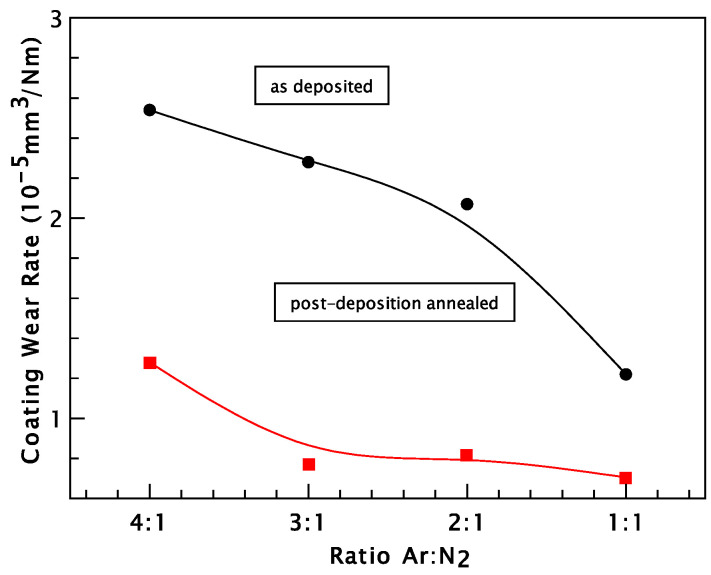
Comparative wear rate of as-deposited CrN and 550 °C annealed coatings (deposited on SS304L stainless-steel substrates).

The comparative wear rates of the CrN and annealed coatings deposited on SS304L stainless-steel substrates are presented in Figure 9. It is evident that the wear rates of the as-deposited CrN coatings, ranging from 1.22 × 10^−5^ to 2.54 × 10^−5^ mm^3^/N·m, are higher than those recorded for the coatings annealed at 550 °C, which range from 1.28 × 10^−5^ to 7.05 × 10^−6^ mm^3^/N·m. This improvement in wear resistance can be attributed to the increase in hardness following annealing.

**Figure 10:**
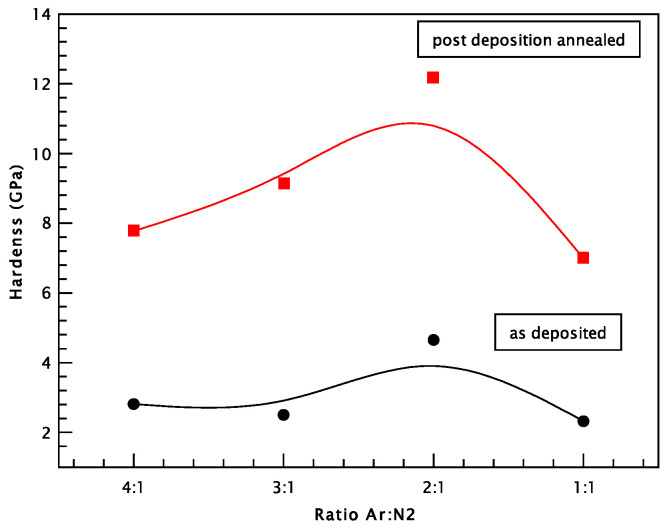
Comparative hardness of as-deposited and 550 °C annealed CrN coatings deposited on SS304L stainless-steel substrates, prepared using various Ar:N_2_ gas ratios during reactive dcMS.

Figure 10 presents the comparative hardness of the as-deposited and post-deposition annealed CrN coatings deposited on SS304L stainless-steel substrates (at 550 °C), prepared using various Ar:N_2_ gas mixtures during reactive dcMS. For the as-deposited CrN coatings, hardness values were relatively low, with measurements of 2.32 GPa for a 1:1 Ar:N_2_ ratio, 2.5 GPa for 3:1, and 2.81 GPa for 4:1. Notably, the highest hardness value among the as-deposited samples, 4.65 GPa, was obtained for the 2:1 gas ratio. All CrN coatings exhibited higher hardness than the stainless-steel substrate (SS304L), which had a measured hardness of 2.10 GPa. The CrN coatings exhibited significantly lower hardness values, between 2.32 and 4.65 GPa, compared to the annealed samples, which reached values between 6.99 and 12.16 GPa.

The low hardness of the material can be attributed to the extremely high dislocation density, on the order of 10^16^ m^−2^. Although the increase in dislocation density is usually correlated with material strengthening (through the dislocation strengthening mechanism), exceeding a critical threshold leads to adverse effects on mechanical properties [49]. Complex interactions between dislocations can lead to the formation of dislocation cells or subgrains, decreasing the resistance to plastic deformation [50]. In addition, the accumulation of a large amount of crystal defects generates a high internal energy that favors structural relaxation processes, such as recrystallization and recovery, with a direct impact on the reduction in hardness [51,52].

The samples annealed at 550 °C exhibit hardness values approximately three times higher than those of the as-deposited coatings. As the nitrogen content decreases, a corresponding increase in hardness is observed, from 6.99 GPa at an Ar:N_2_ gas ratio of 1:1 to a maximum of 12.16 GPa for the 2:1 ratio. Further reduction in nitrogen supply results in decreased hardness values of 9.14 GPa and 7.77 GPa for gas ratios of 3:1 and 4:1, respectively. The significant increase in hardness following annealing at 550 °C may be attributed to the oxidation of chromium and the subsequent formation of the Cr_2_O_3_ phase, which enhances the mechanical properties of the coating [53].

## 4. Conclusions

This research contributed to the development of a fabrication process for CrN-based coatings that combines reactive deposition by dcMS with post-deposition annealing in air, intended for structural applications that subsequently operate in oxidative environments.

In the initial stage, CrN coatings were deposited using reactive dcMS under varying Ar:N_2_ gas ratios 4:1, 3:1, 2:1, and 1:1. The effect of nitrogen supply during deposition was systematically investigated. X-ray diffraction analysis confirmed the formation of CrN phases predominantly for gas ratios of 3:1 and 2:1, while other ratios resulted in structural deviations that may not be optimal for functional applications. Variation in the Ar:N_2_ gas ratio significantly influenced the coating’s structural characteristics (grain size and crystallographic orientation), mechanical properties (hardness), and tribological behavior (coefficient of friction and wear rate).

The post-deposition annealing process further modified the coating structure. Annealing at 550 °C for 1.5 h in air led to the formation of a composite structure comprising both CrN and Cr_2_O_3_ phases, as evidenced by XRD analysis. Notably, samples deposited with an Ar:N_2_ ratio of 2:1 and subjected to annealing exhibited superior performance, achieving the highest hardness value of 12 GPa (approximately three times higher than the as-deposited coatings), the lowest coefficient of friction of 0.254 (approximately three times lower), and a significantly reduced specific wear rate of 8.18 × 10^−6^ mm^3^/N·m (nearly half that of the untreated counterpart).

These results clearly indicate that the structural, mechanical, and tribological properties of CrN-based coatings, characterized by a CrN/Cr_2_O_3_ composite microstructure, are critically dependent on both the deposition process parameters and the post-deposition annealing conditions.

Therefore, by precisely controlling the reactive gas ratio during the dcMS process (from 4:1 to 1:1) alongside the annealing parameters (temperature and duration), the structural, morphological, mechanical, and tribological properties of CrN-based coatings can be effectively optimized for advanced functional applications.

## Figures and Tables

**Table 1:** Measurement parameters for nanoindentation with Oliver and Pharr technique of the SS304L substrate and CrN coatings [35].

Nanoindentation Testing-Measurement Conditions	CrN Coatings	SS304L Steel Substrate
Loading type	linear	linear
Indenter approach speed to the sample (nm/min)	1000	2000
Loading/unloading rate (nm/min)	300	600
Pause at F_max_ (s)	10	10
Data acquisition frequency (Hz)	10	10
Poisson’s ratio (ν)	0.20	0.30

**Table 2:** The thickness of CrN thin films as-deposited using different Ar:N_2_ gas flow ratios.

Sample	N_2_ Flux (sccm)	Ar Flow Rate (sccm)	Film Thickness (nm)	Deposition Rate (nm/min)
CrN4/1	10	40	805	17.88
CrN3/1	12	36	800	17.77
CrN2/1	16	32	680	15.11
CrN1/1	25	25	545	11.11

**Table 3:** XRD data of CrN thin films as-deposited on the NBK-7 glass substrate using different Ar:N_2_ gas flow ratios.

Sample	2θ (Degree)	*(hkl)*	d (Å)	FWHM (Degree)	D (nm)	δ (Lines/m^2^)
CrN4/1	37.74	(111)	2.3816	0.48	16.46	3.69 × 10^15^
CrN3/1	37.66	(111)	2.3866	0.40	19.75	2.56 × 10^15^
CrN2/1	37.69	(111)	2.3847	0.40	19.75	2.56 × 10^15^
CrN1/1	37.69	(111)	2.3850	0.88	8.98	1.26 × 10^16^

**Table 4:** Elemental composition of as-deposited CrN films deposited on silicone substrates as a function of Ar:N_2_ gas ratio.

Sample	Cr (at. %)	N (at. %)	O (at. %)	Si (at. %)
CrN4/1	49.4	29.1	20.2	1.3
CrN3/1	48.4	31.3	19.3	1.1
CrN2/1	47.8	31.4	20.0	0.8
CrN1/1	38.6	38.0	18.2	5.1

**Table 5:** Elemental composition of CrN films annealed at 550 °C as a function of Ar:N_2_ gas ratio.

Sample	Cr (at. %)	N (at. %)	O (at. %)	Si (at. %)
CrN4/1	40.3	3.3	56.2	0.2
CrN3/1	41.2	10.1	48.3	0.4
CrN2/1	38.2	18.8	41.6	1.5
CrN1/1	37.6	18.5	38.6	5.3

## Data Availability

The original contributions presented in this study are included in the article. Further inquiries can be directed to the corresponding author.
